# Lung inflammation perturbation by engineered nanoparticles

**DOI:** 10.3389/fbioe.2023.1199230

**Published:** 2023-05-25

**Authors:** Xiaofei Zhou, Weitao Jin, Jingjun Ma

**Affiliations:** College of Science and Technology, Hebei Agricultural University, Cangzhou, China

**Keywords:** nanoparticles, lung inflammation, mechanisms, physicochemical properties, biosafety evaluation

## Abstract

In recent years, the unique and diverse physicochemical properties of nanoparticles have brought about their wide use in many fields; however, it is necessary to better understand the possible human health risks caused by their release in the environment. Although the adverse health effects of nanoparticles have been proposed and are still being clarified, their effects on lung health have not been fully studied. In this review, we focus on the latest research progress on the pulmonary toxic effects of nanoparticles, and we summarized their disturbance of the pulmonary inflammatory response. First, the activation of lung inflammation by nanoparticles was reviewed. Second, we discussed how further exposure to nanoparticles aggravated the ongoing lung inflammation. Third, we summarized the inhibition of the ongoing lung inflammation by nanoparticles loaded with anti-inflammatory drugs. Forth, we introduced how the physicochemical properties of nanoparticles affect the related pulmonary inflammatory disturbance. Finally, we discussed the main gaps in current research and the challenges and countermeasures in future research.

## 1 Introduction

Owing to their unique physicochemical properties, nanoparticles are widely used in many fields, such as catalysis, optoelectronic devices, energy storage, coatings, environmental protection and biomedicine (Zhou et al., 2020; Ettlinger et al., 2022; Kankala et al., 2022). According to statistics, 5,036 nanoparticls-based products have been officially put on the market by 2023. With the increase in the scale of production and use, nanoparticle-based products will inevitably enter the environment in the process of production, transportation, use, and emission, and they will diffuse into the environment through air, water, food and other media (Iglesias, 2022; Wang et al., 2023). This will increase the chances of nanoparticles entering the human body through respiration, skin contact, food intake and other ways. In addition, nanoparticles used in the field of biomedicine can directly enter the blood circulation system and reach all tissues and organs (Chu et al., 2022; Mundekkad and Cho, 2022). Therefore, the health effects related to human exposure to nanoparticles need to be evaluated urgently.

Lung inflammation is the direct response of the respiratory system to external stimuli. An imbalance in the lung inflammatory response leads to the occurrence of many major lung diseases, such as pulmonary hypertension (Rong et al., 2022), acute lung injury (Zhai et al., 2022), pneumoconiosis (Li et al., 2017), chronic obstructive pulmonary disease (Kim et al., 2023), and lung cancer (Ahmad et al., 2022). 3-Bromopyruvic acid, fucoidan oligosaccharide and astragaloside IV alleviate monocrotaline-induced pulmonary hypertension in rats through an anti-inflammatory pathway (Liu et al., 2020; Jin et al., 2021); The downregulation of miR-let-7e suppresses lung inflammation by targeting the SCOS1/NFκB signaling pathway, thereby reducing acute lung injury induced by lipopolysaccharide (LPS) in mice (Li W. et al., 2021). Ghrelin protects rats from pulmonary vascular dysfunction caused by acute lung injury by inhibiting pulmonary inflammatory response (Li G. et al., 2021); LPS promotes pulmonary fibrosis in silicosis by aggravating the inflammatory response of alveolar macrophages (Tan et al., 2020). The overexpression of myotubularin-related protein 14 (MTMR14) inhibits lung inflammation induced by cigarette smoke extract and improves mitochondrial function. This may be one of the mechanisms by which MTMR14 alleviates chronic obstructive pulmonary disease (Gu et al., 2022). According to the World Health Organization, the death rate due to pulmonary inflammatory response disorders accounts for 75% of the total death rate of acute respiratory infections, which poses a huge threat to health and life of people. To study the effects of high-risk exposure factors on lung inflammation and the related molecular mechanism is of great significance for safeguarding human health and life.

Lung is one of the main target organs of nanoparticles (Montigaud et al., 2020; Scolari et al., 2021). The disturbance of the pulmonary inflammatory response is an important indicator of the pulmonary toxicity of nanoparticles (Guo et al., 2022). Prior studies have shown that exposure to nanoparticles, such as silica nanoparticles (Wang M. X. et al., 2020), titanium dioxide nanoparticles (Ma et al., 2019; Sagawa et al., 2021), and zinc oxide nanoparticles (Guo et al., 2022), interferes with pulmonary inflammatory response in mice, which affects the normal function of the lungs. In this review, we briefly summarize the impact of nanoparticles on lung inflammation from the following four aspects: how nanoparticles activate lung inflammation; how nanoparticles aggravate lung inflammation; how nanoparticles inhibit lung inflammation; and how the physicochemical properties of nanoparticles affect the related lung inflammation disturbance. The challenges and prospects of the disturbance of lung inflammation caused by nanoparticles are also discussed.

## 2 Induction of lung inflammation by nanoparticles

Nanoparticle-induced lung inflammation has been carried out *in vivo* and *in vitro* (Table 1). *In vivo* studies have shown that carbon-based nanoparticles, metal-based nanoparticles, oxide-based nanoparticles, and sulfide-based nanoparticles cause pulmonary inflammatory response in mice or rats after respiratory exposure. First, exposure of C75Bl/6 mice to multi-walled carbon nanotubes (MWCNTs) by intratracheal instillation significantly increases the number of pulmonary macrophages and induces a pulmonary influx of neutrophils and histological analysis has shown the presence of MWCNTs in alveolar macrophages (Luyts et al., 2018). Mesoporous carbon nanoparticles (MCNs) induce biophysical inhibition of the natural pulmonary surfactant, which increases the surface tension of the alveolar, thereby leading to severe alveolar collapse in mice. MCNs also activate macrophages and stimulate lung inflammation associated with lung fibrosis in mice after inhalation exposure (Chen et al., 2017). Similarly, carbon dots (CDs) induce acute lung inflammation and airway macrophages have been identified as target cells of CDs (Weiss et al., 2021). The potential of carbon black nanoparticles (CB nanoparticles) and single-walled carbon nanotubes (SWCNTs) to induce lung inflammation has also been studied in apolipoprotein E-knockout mice (ApoE −/−) and in C57BL/6 J mice. Both SWCNTs and CB nanoparticles significantly increase the expression of IL-6, MIP-2 and MCP-1 mRNA in the lung tissue. They also greatly increases the proportion of neutrophils in bronchoalveolar lavage fluid (BALF) (Jacobsen et al., 2009). Intratracheal administration of SWCNTs remarkably increases the levels of TNF-α, IL-1β, and IL-6 in BALF *via* the activation of the PI3K/AKT/NF-κB signaling pathway (Zhang et al., 2022). A single exposure to graphene oxide (GO) induces lung inflammation by causing DNA damage in the lung alveolar epithelium of C57Bl/6 mice (de Luna et al., 2022). Second, oropharyngeal aspiration of aggregated-MoS_2_ nanosheets induces the neutrophilic exudation into BALF and increases proinflammatory cytokines in C57Bl/6 mice (Wang et al., 2015). After inhalation exposure, PbS nanoparticles induce lung inflammation by causing oxidative stress, thus damaging the blood capillary endothelial cells and alveolar epithelial cells in male Sprague–Dawley rats (Li et al., 2013). Third, following intravenous injection of gold nanoparticles and silver nanoparticles in male Wistar rats, there is an accumulation of gold nanoparticles in the lungs. Histopathological results have shown that infiltrating lymphocytes appear in the lung interstitial tissues, and IL-1α immunostaining is enhanced in the lung tissue, which may be related to the downregulation of miR-327 (Ng et al., 2016). Acute exposure of C57BL/6 mice to Ni nanoparticles elevates the levels of inflammatory factors, IL-6 and CXCL1, along with an increased STAT3 phosphorylation level (You et al., 2020). Intranasal instillation of chitosan-modified Cu nanoparticles also induces lung inflammation in C57BL/6 mice (Worthington et al., 2013). Forth, the widespread use of titanium dioxide nanoparticles (TiO_2_ nanoparticles) as white pigment causes their unintentional release into the environment, which increases the probability of human exposure through the respiratory system. There are more and more studies about the effect of TiO_2_ nanoparticles on lung inflammation. Intratracheal exposure to rutile TiO_2_ nanoparticles resultes in leukocyte migration into alveolar region and significantly increases the secretion of C-C motif ligand (CCL) 3 into BALF. Necrosis inhibitors inhibite the increase of CCL3 secretion in BALF and the increase of leukocytes in BALF. Necrosis of alveolar macrophages that have phagocytosed TiO_2_ nanoparticles is part of the mechanism of acute lung inflammation induced by TiO_2_ nanoparticles (Sagawa et al., 2021). The pulmonary inflammatory response to TiO_2_ nanoparticles shows differences between old and young mice. Compared with old mice, nasal inhalation of TiO_2_ nanoparticles causes more severe lung inflammation and fibrosis in young mice. Decreased levels of global methylation and hydroxymethylation have been found in young mice, in particular, altered methylation in the promoter of TNF-α and Thy-1 have been proven to play a key role in inflammatory response and fibrosis (Ma et al., 2019). Nrf2, a positive modulator of the cytokines IFN-γ, TNF-α and TGF-β, seems to interfere with lung inflammation caused by TiO_2_ nanoparticles exposure (Delgado-Buenrostro et al., 2015). Similarly, Nrf2 also plays a negative regulatory role when zinc-oxide nanoparticles (ZnO nanoparticles) cause the pulmonary inflammatory response (Guo et al., 2022; Sehsah et al., 2022). In both Nrf2 ^−/−^ mice and wild-type mice, the exposure to ZnO nanoparticles increases the number of total cells, lymphocytes, macrophages, and eosinophils in BALF in a dose-dependent manner, but the magnitude of the increase is significantly higher in Nrf2 ^−/−^ mice than in wild-type mice (Sehsah et al., 2019). Silica nanoparticles (SiO_2_ nanoparticles) (Park et al., 2021), nickel-oxide nanoparticles (NiO nanoparticles) (Nishi et al., 2020; Jeong et al., 2022), cobalt-oxide nanoparticles (CoO nanoparticles) (Jeong et al., 2015), and cerium-dioxide nanoparticles (CeO_2_ nanoparticles) (Nemmar et al., 2017) also cause pulmonary inflammatory response in mice or rats. Subchronic intratracheal instillation of Fe_2_O_3_ nanoparticles causes the collagen deposition and infiltration of inflammatory cells *via* the activation of TLR4, TLR2 and downstream myeloid differentiation factor (MyD)88 and NFκB in the lungs of male C57BL/6 mice (Sun et al., 2023). In addition, after exposure through intratracheal instillation, MgO nanoparticles, Cr_2_O_3_ nanoparticles, Co_3_O_4_ nanoparticles, ZnFe_2_O_4_ nanoparticles, NiFe_2_O_4_ nanoparticles, and NiZnFe_4_O_8_ nanoparticles also cause inflammation in female Wistar rats or female C57BL/6 mice (Cho et al., 2012; Hadrup et al., 2020).

**TABLE 1:** Lung inflammation induced by nanoparticles.

Nanoparticles	Cell lines/animal model	Administration	Dose	Mechanisms	Induction/aggregation	Ref
Mesoporous carbon nanoparticles	Female BALB/c mice	Pharyngeal aspiration	50 mg/kg	ROS	Induction	Chen et al. (2017)
Carbon dots	Male BALB/c mice	Intranasal instillation	100 μg	ROS	Induction	Weiss et al. (2021)
MWCNTs	Male C57Bl/6 mice	Intratracheal instillation	512 μg/mL (25.6 μg/instillation)	Coagulation factor VIII	Induction	Luyts et al. (2018)
MWCNTs	C57BL/6 mice	Oropharyngeal aspiration	2 mg/kg	STAT6	Induction	Shipkowski et al. (2015)
SWCNTs	Female C57BL/6 mice	Oropharyngeal aspiration	40 μg/mouse	PI3K/AKT/NFκB	Induction	Zhang et al. (2022)
CB nanoparticles	RLE-6TN, C57BL/6 mice	Pharyngeal aspiration	10 μg/mL	Ceramides, EGFR	Induction	Peuschel et al. (2012)
CB nanoparticles	A549	—	25 μg/mL	PKC-α	Induction	Hsu et al. (2018)
GO	Female C57BL/6 mice	Intraperitoneal injection	30 μg	DNA damage	Induction	de Luna et al. (2022)
Au nanoparticles	Male Wistar rats	Intravenous administration	0.2 mg/kg	miR-327	Induction	(Ng et al., 2016)3
Ag nanoparticles	Male Wistar rats	Intravenous administration	0.2 mg/kg	miR-327	Induction	Ng et al. (2016)
Ag nanoparticles	Male Fischer rats	Inhalation	179 μg/m^3^, 6 h per day for 4 days		Induction	Braakhuis et al. (2014)
Cu nanoparticles	Male C57BL/6 mice	Intranasal instillation	30 μg/mouse		Induction	Worthington et al. (2013)
Ni nanoparticles	C57BL/6 mice	Oropharyngeal aspiration	4 mg/kg	STAT3	Induction	You et al. (2020)
SiO_2_ nanoparticles	C57BL/6 mice	Intratracheal instillation	10 mg/kg	ROS, PARP, TRPM2	Induction	Wang et al. (2020b)
SiO_2_ nanoparticles	MRC-5	—	62.5 μg/mL	NFκB	Induction	Voicu et al. (2019)
SiO_2_ nanoparticles	Female C57BL/6 mice, J774 macrophages	—	2.5 mg/kg	IL-1α	Induction	Rabolli et al. (2014)
SiO_2_ nanoparticles	Male ICR mice	Intratracheal instillation	50 μg/mouse	Apaf-1, caspase-3	Induction	Park et al. (2021)
NiO nanoparticles	BEAS-2B, A549	—	100 μg/mL	NFκB, MAPK	Induction	Capasso et al. (2014)
NiO nanoparticles	Male Wistar rats	Intratracheal instillation	0.2 mg (0.66 mg/kg)	Alveolar macrophages damage	Induction	Nishi et al. (2020)
NiO nanoparticles	Female Wistar rats	Intratracheal instillation	150 cm^2^/rat	Perturbation of lung microbiome	Induction	Jeong et al. (2022)
ZnO nanoparticles	A549, Hacat	—	35 μg/mL	ROS	Induction	Almutairi et al. (2020)
ZnO nanoparticles	Female C57BL/6 mice	Intratracheal instillation	10 μg, 20 μg	Nrf2	Induction	Sehsah et al. (2019), Guo et al. (2022), Sehsah et al. (2022)
ZnO nanoparticles	Male C57Bl/6 mice	Intratracheal instillation	256 μg/mL (12.8 μg/instillation)	Coagulation factor VIII	Induction	Luyts et al. (2018)
CdO nanoprticles	Male CD1 mice	Inhalation	1.7 μg	Matrix metalloproteinases (MMP)-2, MMP-9	Induction	Blum et al. (2014)
CeO_2_ nanoparticles	Female BALB/c mice	Intratracheal instillation	0.5 mg/kg	ROS, DNA damage	Induction	Nemmar et al. (2017)
CoO nanoparticles	Female rats	Intratracheal instillation	400 μg/rat	Neutrophil influx	Induction	Jeong et al. (2015)
Co_3_O_4_ nanoparticles	Female rats	Intratracheal instillation	400 μg/rat	Neutrophil influx	Induction	Jeong et al. (2015)
Fe_2_O_3_ nanoparticles	Male C57BL/6 mice	Intratracheal instillation	300 μg/mouse	TLR2, TLR4, MyD88, TRAF6, NFκB	Induction	Sun et al. (2023)
TiO_2_ nanoparticles	Male NIH mice	Intranasal instillation	20 mg/kg	Methylation of TNF-α, Thy-1	Induction	Ma et al. (2019)
TiO_2_ nanoparticles	C57BL/6 mice	Inhalation	5 mg/kg	Nrf2	Induction	Delgado-Buenrostro et al. (2015)
TiO_2_ nanoparticles	C57BL/6 mice	Intratracheal instillation	200 μg	C-C motif ligand (CCL) 3	Induction	Sagawa et al. (2021)
Al_2_O_3_ nanoparticles	Male Wistar rats	Intranasal instillation	20.0–22.1 mg/m^3^, 24 h	ROS, DNA damage	Induction	Bourgois et al. (2021)
MoS_2_ nanoparticles	C57BL/6 mice	Oropharyngeal aspiration	2 mg/kg	LIX, MCP-1	Induction	Wang et al. (2015)
PbS nanoparticles	Male Sprague–Dawley rats	Inhalation	30 mg/kg	ROS	Induction	Li et al. (2013)
CB nanoparticles	Male ICR mice	Intratracheal instillation	4 mg/kg	ROS	Aggravation	Inoue et al. (2006)
MWCNTs	Male Sprague-Dawley rats	Intratracheal instillation	4 mg/kg	PDGF	Aggravation	Cesta et al. (2010)
MWCNTs	Male B6C3F1/N mice	Inhalation	0.6 mg/m^3^, 30days	Th2 cytokines	Aggravation	Ihrie et al. (2019)
SiO_2_ nanoparticles	Female BALB/c mice	Intranasal instillation	10 mg/kg	Airway hyper-responsiveness	Aggravation	Han et al. (2016)
TiO_2_ nanoparticles	BALB/c mice	Inhalation	50 μg/m^3^, 3days	ROS, NLRP3	Aggravation	Kim et al. (2017a)
ZnO nanoparticles	Female Balb/c mice	Oropharyngeal aspiration	0.5 mg/kg	Th2 cytokines	Aggravation	Huang et al. (2015)

*In vitro* studies have also proven that nanoparticles induce lung inflammation by activating various cell signaling pathways. TRPM2, IL-1α, NFκB, PKC-α, and EGFR participate in the inflammatory response caused by nanoparticles in lung cells (Peuschel et al., 2012). In BEAS-2B cells, SiO_2_ nanoparticles cause an increase in ROS production, the activation of TRPM2 channel, and the alteration of intracellular Zn^2+^ and Ca^2+^ homeostasis mediated by TRPM2, thereby resulting in lysosome impairment and subsequent blockade of autophagy flux. The abnormal autophagy triggers the production of proinflammatory mediators, leading to lung inflammation (Wang M. X. et al., 2020). Exposure to SiO_2_ nanoparticles causes the rapid release of IL-1α from the preexisting reserve in alveolar macrophages and stimulates subsequent lung inflammation through the production of IL-1β. Further, the release of IL-1α can be used to predict the induction of acute lung inflammation (Rabolli et al., 2014). Amorphous negatively charged SiO_2_ nanoparticles induce the production of proinflammatory markers by upregulating NFκB and reducing the activity of MMP in MRC-5 lung fibroblasts (Voicu et al., 2019). A similar mechanism has been found in A549 cells exposed to NiO nanoparticles. NiO nanoparticle-induced proinflammatory cytokines are dependent on the mitogen-activated protein kinases (MAPK) cascade *via* the activation of the NFκB pathway (Capasso et al., 2014). In A549 cells, CB nanoparticles induce the activation of PKC-α and significantly increase the secretion of inflammatory factors, including COX-2, NO, iNOS and PGE(2). PKC-α inhibitor reduces CB nanoparticle-induced inflammation by downregulation of NO, PGE(2), and ROS, which indicates that PKC-α might participate in CB nanoparticle-induced inflammation (Hsu et al., 2018). In short, there are a large number of receptors or proteins that regulate inflammation on the cell surface or intracellularly, such as TLR4 (Cao et al., 2023), TNFR (McDaniel et al., 2022), P2X7R (Jin et al., 2017), cathepsins (de Mingo et al., 2016), and caspase-1 (Flores et al., 2022). Intracellular inflammation-related signaling pathways are very complex. Thus, the molecular mechanism of lung inflammation induced by nanoparticles is still in its infancy, and there are still numerous unknown signaling proteins to be examined in further research.

Existing studies have proven that the exposure to a variety of traditional nanoparticles causes lung inflammation in mice, rats, and other experimental animals. Due to the increasingly mature synthesis methods of nanoparticles, novel nanoparticles, such as two-dimensional transition metal dichalcogenides (Kirubasankar et al., 2022), black phosphorus nanoflakes (Wang et al., 2021), and metal-organic framework nanoparticles (Chen et al., 2022), have begun to enter the market. However, it is not yet completely clear whether the exposure to these novel nanoparticles can cause lung inflammation. To understand the lung health risks of nanoparticles, it is necessary to fully clarify the disturbance of lung inflammation by nanoparticles and the related molecular mechanisms.

## 3 Aggravation of lung inflammation by nanoparticles

When different types of lung inflammation occur, the exposure to nanoparticles can further aggravate the inflammatory response. First, after acute lung inflammation caused by LPS in rats or mice, nanoparticle treatment aggravates the existing inflammatory response through various pathways. The glycolipids of Gram-negative bacteria and LPS stimulate host cells through innate immunity. In animal models, intratracheal instillation of LPS can cause lung neutrophil recruitment, lung cytokine expression, and lung injury. When rats are exposed to LPS and then treated with MWCNTs intratracheally for 24 h, it is obvious that LPS alone does not cause lung fibrosis, but the co-treatment of LPS and MWCNTs enhances pulmonary fibrosis. The reason may be that MWCNTs increase the level of platelet-derived growth factor-AA (PDGF-AA), the main mediator of fibrosis. LPS cooperatively enhances the PDGF-AA generation by MWCNTs. *In vitro* experiments in rat lung macrophages (NR8383 cells) and rat lung fibroblasts have also verified that LPS exposure enhances the mRNA level of PDGF-AA induced by MWCNTs. That is, LPS aggravates MWCNT-induced pulmonary fibrosis by increasing the production of PDGF-AA in macrophages and epithelial cells, and by amplifying PDGF-AA on lung fibroblasts (Cesta et al., 2010). Fourteen-nanometer CB nanoparticles significantly aggravate LPS-induced lung inflammation and pulmonary edema, accompanied by the increased pulmonary expression of macrophage inflammatory protein-1α (MIP-1α), IL-1β, keratinocyte chemoattractant, macrophage chemoattractant protein-1 and MIP-2 (Inoue et al., 2006). Intratracheal instillation of TiO_2_ nanoparticles (Inoue et al., 2008) nanoparticles and ZnO nanoparticles (Wang P. et al., 2020) into mice further aggravates LPS-induced pulmonary inflammatory response in mice by enhancing the expression of proinflammatory cytokines and chemokines, promoting oxidative stress, and causing DNA damage and cell apoptosis. Second, nanoparticles aggravate the pulmonary inflammatory response caused by ovalbumin or dust mites. TiO_2_ nanoparticles treatment exacerbates ovalbumin-induced lung inflammation in mice, which may be due to the increased ROS level, enhanced expression of IL-18 and IL-1β, and activation of NLRP3 inflammasome (Kim B.-G. et al., 2017). Similarly, intranasal administration of spherical SiO_2_ nanoparticles aggravates ovalbumin-induced allergic airway inflammation in mice (Han et al., 2016). Inhalation exposure to MWCNTs aggravates the pulmonary inflammatory response caused by dust mites (Shipkowski et al., 2015; Ihrie et al., 2019). In short, the existing studies have confirmed that the respiratory system exposure to nanoparticles exacerbates the ongoing lung inflammatory response. There are different types of lung inflammation, including LPS- or ovalbumin-induced lung inflammation. The effect of nanoparticles on lung inflammation may be related to the specific type of lung inflammation. There is an urgent need for in-depth research to clarify this issue.

## 4 Inhibition of lung inflammation by nanoparticles

**FIGURE 1:**
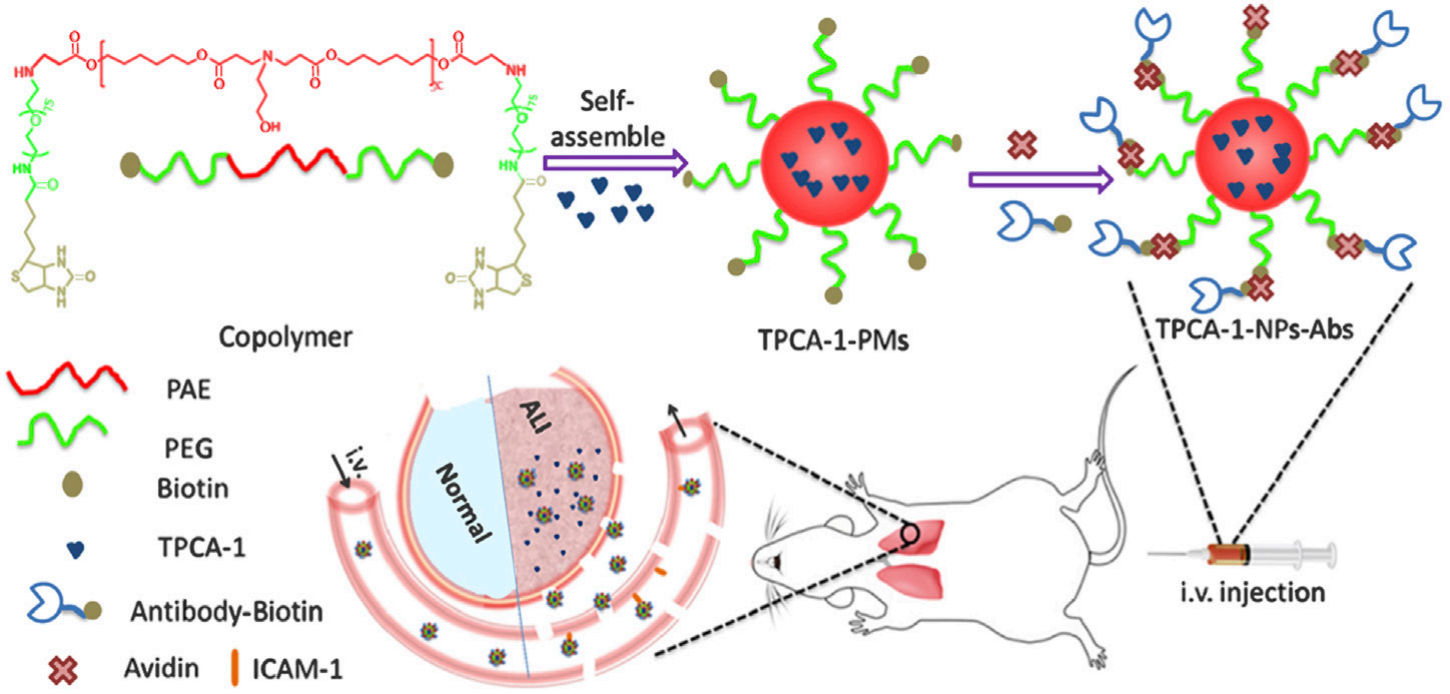
Illustration of the development of Ab-decorated nanoparticles targeted to mouse lungs for the treatment of acute lung inflammation (Zhang et al., 2019). Copyright ^©^ 2019, American Chemical Society.

Recent studies have verified that nanoparticles loaded with special drugs inhibit lung inflammation. Lipid nanoparticles loaded with cepharanthine and coated with macrophage membrane (Lu et al., 2021), dexamethasone-loaded ROS-responsive poly (thioketal) nanoparticles (Zhai et al., 2022), nanoparticles containing dexamethasone modified with hyaluronic acid (Camara et al., 2021), neutrophil membrane-coated, antibiotic agent-loaded nanoparticles (Wang K. Y. et al., 2020), platelet vesicle-decoyed poly (lactic-co-glycolic acid) nanoparticles (Jin et al., 2022), silymarin/curcumin-loaded albumin nanoparticles coated with chitosan (Hanafy and El-Kemary, 2022), shell-crosslinked-knedel-like nanoparticles (Ibricevic et al., 2013), and bilirubin nanoparticles (Kim D. E. et al., 2017) inhibit lung inflammation in mice. Nanoparticles loaded with multiple drugs inhibited lung inflammation *via* different molecular mechanisms. For example, bixin-loaded polymeric nanoparticle treatment significantly reduces the number of leukocytes and TNF-α level, and it strongly inhibits the increase of MDA and PNK in lung homogenates in BALF of mice exposed to cigarette smoke. The beneficial effect may be attributed to the ability of bixin to clear and neutralize oxidative substances and block the harmful continuous events caused by cigarette smoke (Figueiredo-Junior et al., 2022). Fluorous-tagged peptide nanoparticles significantly ameliorate LPS-induced acute lung inflammation by maintaining the stability of lysosomal membrane and increasing the expression levels of Nrf2, NQO1, and HO-1 (Wang et al., 2022). The pulmonary deposition of CeO nanoparticles alleviates the lung inflammation induced by hypobaric hypoxia by inhibiting the formation of ROS, lipid peroxidation, and glutathione oxidation, and preventing the oxidative modification of proteins (Arya et al., 2013). A new pH-responsive drug-delivery system, TPCA-1-loaded nanoparticles coated with anti-ICAM-1, selectively targets inflammatory endothelium and mouse lungs after intravenous injection, and then the acid environment triggers drug release, thereby reducing lung inflammation and injury (Figure 1) (Zhang et al., 2019). In short, due to the great specific surface area and surface modifiability, the surface of nanoparticles can be modified with targeting molecules and anti-inflammatory drugs, so as to achieve effective inflammatory treatment by targeting specific inflammatory sites, which is a very promising idea for the treatment inflammatory diseases.

Persistent inflammatory conditions can induce DNA damage and mutations, thereby increasing cell division rate damage in lung tissue. Generally, lung cancer usually occurs in the inflammatory tumor microenvironment (Rajasegaran et al., 2023). The occurrence of lung cancer is related to various factors that cause lung inflammation, such as IL-1 *β*. *In vitro* and *in vivo* data have indicated that IL-1β in particular promotes the migration and invasion of lung cancer cells, triggering more aggressive cancer phenotypes (Gelfo et al., 2020; Zhang et al., 2020) (Voronov et al., 2003; Das et al., 2020). Inhibiting the expression of IL-1β has been shown to reduce tumor growth and prevent the shedding of tumor cells from the primary site into circulation (Kaplanov et al., 2019; Tulotta et al., 2019; Zhang and Veeramachaneni, 2022). In a study of 28 advanced non-small-cell lung cancer patients, plasma IL-1β level was increased (McLoed et al., 2016). Another study demonstrated that the inhibition of IL-1β in combination with 5-fluorouracil overcame the resistance mechanisms and enhanced the antitumor function (Bruchard et al., 2013). Some nanoparticles loaded with drugs can effectively inhibit lung inflammation caused by LPS and allergens, thereby reducing the level of inflammatory factor IL-1β. Considering the important role of inflammatory conditions and IL-1β in the occurrence and development of lung cancer, we hope that the inhibition of IL-1β induced by these nanoparticles will open new avenues for cancer treatment by targeting lung tumor inflammation.

According to their composition, nanoparticles can be divided into hard nanoparticles and soft nanoparticles. Metal-based nanoparticles, metal-oxide nanoparticles, and carbon-based nanoparticles belong to hard nanoparticles, while liposome nanoparticles and polymer nanoparticles belong to soft nanoparticles. It is generally believed that soft nanoparticles are less toxic than hard nanoparticles due to their biodegradability. When focusing on the toxic effects of disturbing the pulmonary inflammatory response, hard nanoparticles are more likely to cause and exacerbate lung inflammation. For example, CNTs, MCNs, CDs, CB nanoparticles (Inoue et al., 2006; Jacobsen et al., 2009), TiO_2_ nanoparticles, ZnO nanoparticles, CoO nanoparticles (Huang et al., 2015; Jeong et al., 2015; Ma et al., 2019), NiO nanoparticles, SiO_2_ nanoparticles, CeO_2_ nanoparticles, and gold nanoparticles (Bachand et al., 2012; Han et al., 2016; Nemmar et al., 2017; Nishi et al., 2020) induce or exacerbate lung inflammation in mice, rats, or lung cells. In contrast, soft nanoparticles loaded with drugs, such as lipid nanoparticles (Lu et al., 2021), polymeric nanoparticles (Ibricevic et al., 2013; Zhang et al., 2019; Wang K. Y. et al., 2020; Figueiredo-Junior et al., 2022; Jin et al., 2022; Zhai et al., 2022), protein nanoparticles (Hanafy and El-Kemary, 2022), bilirubin-based nanoparticles (Kim D. E. et al., 2017), and nanoparticles with a shell of hyaluronic acid and a core of dexamethasone (Camara et al., 2021) are more likely to inhibit lung inflammation.

The conclusion that nanoparticles cause or aggravate lung inflammation is mostly drawn from the research of nanoparticles without drug loading, and the conclusion that nanoparticles inhibit lung inflammation is mainly drawn from the research of nanoparticles loaded with special drugs. There are relatively few studies on the inhibition of lung inflammation after exposure to nanoparticles alone, and the specific molecular mechanism remains to be further studied. In order to fully understand the interference of nanoparticles with lung inflammation, it is necessary to clarify the effects and related molecular mechanisms of nanoparticle exposure when lung inflammation has already occurred.

## 5 Roles of nanoparticles characteristics on the regulation of lung inflammation

The disturbance of lung inflammation caused by nanoparticles is closely related to their physicochemical properties. First, research based on CB nanoparticles, TiO_2_ nanoparticles, and silica-dioxide nanoparticles has confirmed that the smaller the particle size of nanoparticles, the easier it is to cause or aggravate lung inflammation. When the weight of nanoparticles is equal, the airway exposure to 14-nm CB nanoparticles strongly aggravates LPS-induced pulmonary edema and lung inflammation, while 56-nm nanoparticles do not show obvious effects (Inoue et al., 2006). Next, 20-nm silica nanoparticles, but not 50-nm silica nanoparticles, induce lung inflammation in rats after repeated exposure for 14 days. Compared with the cells treated with 50-nm silica nanoparticles, the structural damage of organelles in the cells treated with 20-nm silica nanoparticles is more obvious, and the increase of mitochondrial membrane potential and mitochondrial calcium accumulation is only observed in 20-nm silica nanoparticle-treated cells. The lung inflammation induced by 20-nm silica nanoparticles may be related to the paraptosis of alveolar macrophages (Park et al., 2021). Three sizes (15, 50, and 100 nm) of TiO_2_ nanoparticles aggravate LPS-induced lung inflammation and vascular permeability in a size-dependent manner after 24 h of intratracheal instillation in mice. Compared with LPS alone, LPS plus silica nanoparticles, especially those smaller than 50 nm in size, improve the circulatory level of MCP-l, fibrinogen, KC, IL-l p and von Willebrand factor (Inoue et al., 2008). This may be because the smaller size of nanoparticles make it easier for them to enter the lungs. Second, the surface modification of nanoparticles influences many biological effects, such as autophagy, apoptosis, and oxidative stress (Zhou et al., 2022). As an immune response to exogenous substances, lung inflammation is also interfered by the surface modification of nanoparticles. Coating copper-oxide nanoparticles with chitosan reduces their ability to be removed from the lungs, prolongs the exposure time of lung cells and tissues to metal oxides, and produces significant acute lung inflammation (Worthington et al., 2013). This may be attributed to the fact that the surface modification may change the charge, hydrophobicity and steric hindrance of nanoparticles, thereby affecting the cellular uptake, subcellular localization of nanoparticles, and interaction between nanoparticles and cell surface proteins (Sun et al., 2018; Bai et al., 2020). Solubility and thickness also affect the disturbance of lung inflammation caused by nanoparticles (Cho et al., 2012; Wang et al., 2015). Forty hours after oropharyngeal aspiration in C57Bl/6 mice, thick aggregated-MoS_2_ nanosheets induce robust production of IL-6, MCP-1, and LIX along with the neutrophilic exudation into BALF, whereas thin MoS_2_ nanosheets do not trigger chemokine or cytokine induction in the lungs. Histopathological changes confirme the formation of focal areas of inflammation around small airways induced by thick aggregated-MoS_2_ nanosheets, while thin MoS_2_ nanosheets have little or no effect (Wang et al., 2015). After 24 h of intratracheal instillation, high-solubility CoO nanoparticles produce a dose-dependent eosinophilic influx into the lungs. The inflammatory potential of CoO nanoparticles is comparable to that evaluated after treatment with an identical Co. ion mass of CoCl_2_, while the medium-solubility Co_3_O_4_ nanoparticles do not induce the eosinophilic inflammation. Eosinophilic inflammation produced by CoO nanoparticles might originate from the dissolution of Co. ions inside the cells (Jeong et al., 2015). The physicochemical properties of nanoparticles affect their biological effects. We speculate that the shape, composition and surface protein corona of nanoparticles also influence their disturbance of lung inflammation. Further research is urgently needed to clarify the specific link between these physicochemical properties and lung inflammation.

## 6 Discussion

This review summarized the activation of lung inflammation caused by nanoparticles, the aggravation of lung inflammation caused by nanoparticles, the inhibition of lung inflammation caused by nanoparticles, and the influence of physicochemical properties of nanoparticles on the disturbance of lung inflammation. Due to unique physicochemical properties and increasingly mature synthesis methods, nanoparticles have widely been used in many fields, thereby increasing the opportunities for human exposure. Once nanoparticles enter the human body, they may interact with the biological system, disturb the steady state of the physiological system, and pose a threat to human health. Therefore, it is necessary to evaluate the biological safety of nanoparticles. As the lungs are an important target organ of nanoparticles, it is significant to evaluate the disturbance of lung inflammation by nanoparticles. The relevant fields are still in the initial stage, and there are many key issues that still need to be studied in depth.

From the perspective of lung inflammation, a large number of studies have focused on how nanoparticles trigger or aggravate lung inflammation. The conclusions are mainly drawn from studies of nanoparticles without drug loading. In contrast, limited studies have found that nanoparticles loaded with anti-inflammatory drugs inhibit the ongoing lung inflammation. Research about the inhibition of lung inflammation due to an individual’s exposure to drug-free nanoparticles is still in its infancy.

Existing studies have shown that the physicochemical properties (size, shape, composition, and surface chemistry) of nanoparticles affect their interaction with biological systems. However, research on the influence of the physicochemical properties of nanoparticles on the disturbance of lung inflammation is still in its infancy. Limited studies have preliminarily found that size, shape, and surface charge may affect the disturbance of lung inflammation caused by nanoparticles. Therefore, it is necessary to systematically study how various physicochemical properties affect the disturbance of lung inflammation caused by nanoparticles and the specific molecular mechanism. The ultimate goal of the lung safety assessment of nanoparticles is to reveal the potential risks of nanoparticles to human lung health. The conclusions obtained *in vitro* need to be further verified by *in vivo* experiments. The specific relationship between the physicochemical properties and the disturbance of lung inflammation caused by nanoparticles should be clarified through the systematic study at the body level.
